# Inhibition of *α*-Amylase and *α*-Glucosidase of Anthocyanin Isolated from *Berberis integerrima* Bunge Fruits: A Model of Antidiabetic Compounds

**DOI:** 10.1155/2022/6529590

**Published:** 2022-03-07

**Authors:** Soheila Moein, Mahmoodreza Moein, Hassan Javid

**Affiliations:** ^1^Medicinal Plants Processing Research Center, Shiraz University of Medical Sciences, Shiraz, Iran; ^2^Department of Pharmacognosy, School of Pharmacy, Shiraz University of Medical Sciences, Shiraz, Iran; ^3^BiochemistryDepartment, Faculty of Medicine, Hormozgan University of Medical Sciences, Bandarabbas, Iran

## Abstract

Anthocyanins are components of the flavonoid group with different properties, such as antidiabetic properties. This study aimed to isolate anthocyanin from *Berberis integerrima* Bunge fruits and evaluate *α*-amylase and *α*-glucosidase inhibition by this mentioned anthocyanin. The anthocyanin of *Berberis integerrima* fruit was isolated using column chromatography, and the antidiabetic properties of the anthocyanin were determined by the levels of *α*-amylase and *α*-glucosidase inhibition. Km and Vmax were also evaluated using the GraphPad Prism 7. The results of this study showed that the anthocyanin content of the fruit extract was 14.36 ± 0.33 mg/g, and following purification, this amount increased to 34.51 ± 0.42 mg/g. The highest of *α*-glucosidase inhibition was observed in the purified anthocyanin with IC_50_ = 0.71 ± 0.085 mg/ml, compared to acarbose as the baseline with IC_50_ = 8.8 ± 0.14 mg/ml, *p* < 0.0001. Purified anthocyanin of the mentioned fruit with IC_50_ = 1.14 ± 0.003 mg/ml had the greatest *α*-amylase inhibition, which was similar to acarbose as the standard with IC_50_ = 1 ± 0.085 mg/ml, *p* < 0.05. The inhibition of *α*-glucosidase and *α*-amylase by purified anthocyanin showed uncompetitive inhibition, and the enzyme inhibition by unpurified anthocyanin showed mixed inhibition. The obtained findings showed that *Berberis integerrima* fruit can be mentioned as a source of anthocyanin with antidiabetic properties.

## 1. Introduction

The characteristics of the metabolic syndrome such as diabetes include high levels of glucose, obesity of the abdomen, and high level of cholesterol and high blood pressure, which increase the risk of cardiovascular disease. Insulin resistance is a significant feature of metabolic syndrome and type 2 diabetes. Nowadays, there are 246 million patients with type 2 diabetes in the world. The International Diabetes Federation (IDF) announced that 380 million people will be diabetic in 2025 [1]. Some plant extracts and plant compounds can reduce blood sugar and prevent diabetes.

In folk medicine, barberry fruits are consumed as an antidiabetic drug [2], which composed of great amounts of anthocyanin [3]. Anthocyanin pigments are globally well-known as medicinal agents.

Free radical scavengers are the most widely known properties of anthocyanins [3].

In addition, it was reported that the anthocyanin content of barberry fruits is similar to the anthocyanin content of blackberries fruits (*Rubus glaucus*) [4], nasturtium (*Tropaeolum majus*) [5], and a hybrid of strawberries (*Fragaria ananassa*) [6].

Some studies [7] showed that, in vitro, anthocyanins stimulate insulin secretion from the rodent pancreatic *β*-cells. Besides, consumption of fruits and vegetables rich in polyphenols reduces the incidence of type 2 diabetes [8, 9].

Anthocyanin compounds are abundant in food plants and appear in 27 plant families. The usage of anthocyanin has been estimated at 10000 tons of black grapes in one year. Anthocyanins are vital components of plant secondary metabolites and are also the most important coloring substances in wine [10].

The six anthocyanins usually presented in plants are classified according to the position and number of hydroxyl groups on the flavan nucleus and are nominated as cyanidin (cy), delphinidin (dp), malvidin (mv), peonidin (pn), pelargonidin (pg), and petunidin (pt). Anthocyanins are water-soluble glycosides of polyhydroxyl and polymethoxyl derivatives of 2-phenylbenzopyrylium or flavylium salts. The discrimination between anthocyanins is based on the position and number of hydroxyl groups; the level of methylation of these hydroxyl groups; the number, nature, and location of sugars attached to the molecule; and aromatic or aliphatic acids attached to the sugars in the molecule. The glycosylation increases water solubility and structural stability of the anthocyanidin [11].

Another study reported that, in vitro, the whole extract or pure form of anthocyanin has antioxidant properties, higher than the other natural antioxidants [12–14].

The purpose of this study was to evaluate the anthocyanin antidiabetic properties of *Berberis integerrima* Bunge fruits (BIBF) before loading on to the column chromatography and after being isolated from the column. For evaluation, the antidiabetic properties of BIBF, *α*-amylase, and *α*-glucosidase inhibition were assessed.

## 2. Materials and Methods

### 2.1. Chemicals

PNPG (4-nitrophenyl *α*-d-glucopyranoside), 3, 5-dinitro salicylic acid (DNSA), and *α*-glucosidase and *α*-amylase enzymes were obtained from Sigma Aldrich, USA. Other substances were purchased from Merck Chemicals (Darmstadt, Germany).

### 2.2. Plant Materials

*Berberis integerrima* Bunge was gathered from Kohmar (around Shiraz, capital of Fars province, Iran) in September 2018 and was identified and authenticated (Voucher No. Pm396) in the Medicinal Plants Museum, Department of Pharmacognosy, University of Medical Sciences, Shiraz, Iran [15].

### 2.3. Extraction and Purification

Three hundred and twenty-two grams of the frozen-dried fruit powder was percolated with ethanol, and the obtained extract was concentrated under vacuum in the rotary evaporator, followed by a speed vacuum to yield 140 g of the gummy substance. Thirty grams of crude extract was then suspended in 0.3% TFA. Afterward, it was filtered and mixed with ethyl acetate. Then, the liquid phase was gathered (this extraction repeated three times); this phase was packed on to the Amberlite column (2.5 × 45 cm). Following that, the column was washed out with distilled water containing 0.3% of TFA to discharge polysaccharides. The purified anthocyanin was eluted with 0.3% of TFA dissolved in methanol and then concentrated by the rotary evaporator [15]. The amount of obtained extracts and anthocyanin fraction were 20% and 2.57%, respectively [15].

### 2.4. Measurement of the Anthocyanin Content

A pH-differential was applied to determine the anthocyanin content in the BIBF fraction. 80.4 ml of the extract was combined with 3.6 ml of the buffer. The buffer was a mixture of 0.4 M sodium acetate solution and 0.025 M potassium chloride solution, and the buffer was fixed at pH 4.5. The absorbance of each sample was determined at 519 nm, and the distilled water was used as a blank. The anthocyanin content was calculated via the following equation:

(1) $$\begin{array}{c} \text{Anthocyanin content}\left(\frac{\text{mg}}{\text{ml}}\right)=\frac{\left(A\times \text{MW}\times \text{DF}\times 1000\right)}{\varepsilon \times L}, \end{array}$$

where A is (A519 (pH 1.0)-A519 (pH 4.5)), MW is the molecular weight of anthocyanin (433.2 g/mol), DF is the dilution factor, *ε* is the extinction coefficient (31600 L cm^−1^mol^−1^), and *L* is the path length (1 cm) [15].

### 2.5. Determination of the Inhibition of *α*-Glucosidase

Inhibition of *α*-glucosidase was assessed according to the method reported by McCue et al. [16]. The enzyme solution included 125 *μ*l of phosphate buffer (pH 6.9, 0.1 M) and 5 *μ*l of *α*-glycosidase (25 unit/ml). In addition, 11 mM of P-nitrophenyl-*α*-D-glucopyranoside dissolved in the phosphate buffer (pH 6.9) was applied as a substrate solution. Then, 20 *μ*l of the sample at various concentrations was mixed with the enzyme solution and incubated for 15 min at 37°C. The reaction was initiated by the addition of 20 *μ*l of the substrate solution and incubation for an extra 15 min. The addition of sodium carbonate (0.2 M, 80 *μ*l) retarded the reaction.

The absorbances of the samples were determined at 405 nm using a microplate reader, while the control did not contain the sample. The blank did not contain *α*-glucosidase, and acarbose was used as the standard sample. All the evaluations were performed three times. Finally, the enzyme inhibition rates of the samples were determined via the following equation:

(2) $$\begin{array}{c} \text{Inhibition \%}=\left[\frac{\left(\text{control absorption}-\text{sample absorption}\right)}{\text{control absorption}}\times 100\right]. \end{array}$$

### 2.6. Kinetics of *α*-Glucosidase Inhibition

The inhibition modes of *α*-glucosidase by the samples were assessed based on the method reported by Kim et al. [17]. In doing so, fixed amounts of *α*-glucosidase were blended with increasing concentrations of its substrates (PNPG) at 37°C for 15 min without and with the IC_50_ concentrations of the samples. After that, the reactions were stopped, and absorption was determined using the Michaelis–Menten curve. All the evaluations were repeated three times.

### 2.7. Determination Inhibition of *α*-Amylase

Inhibition of *α*-amylase (EC:3.2.1.1) by different concentrations of the samples according to the method reported by Maccu and Shetty was determined [16]. In this study, acarbose was used as a standard. First, the samples were mixed with distilled water and starch (as a substrate). The reaction started with the addition of *α*-amylase. Then, the mixture was incubated for 15 min at 37°C, and after that, dinitro salicylate alkaline potassium tartrate was added. Afterward, another incubation was performed for 15 minutes at 85°C, and finally, the activity of *α*-amylase was evaluated by determination of the absorbance at 540 nm (Biotek Corporation's synergy HTX multimode reader). The control contained DMSO (dimethyl sulfoxide) instead of the sample. Blank contained buffer instead of the enzyme. For evaluating the mode of *α*-amylase inhibition, the IC_50_ concentration of the sample and different concentrations of the starch were used according to the method mentioned previously. After obtaining the absorption rates for all of the substrate concentrations, the absorbance vs. substrate concentration was drawn until this curve reached saturation or plateau, indicating the maximum velocity.

### 2.8. Statistical Analysis

The Tukey post hoc test was used to compare the IC_50_ means of the samples and the *P* value.

If the *P* value was <0.05, then the difference is considered as significant.

First, we converted the amount of sample absorption into enzyme activity using the product molar extinction coefficient. Then, we entered the values of the substrate concentrations and the activity level of the enzyme into the Graphpad Prism software, which calculated the values of Km and *V*_max_. After that, the mode of inhibition was evaluated.

## 3. Results and Discussion

In an in vivo study, it was significantly confirmed that dietary consumption of blueberry and black raspberry anthocyanin protects blood cells from oxidative stress and free radicals [18, 19]. Therefore, it shows the vital role of anthocyanin against reactive oxygen species [20].

The amount of anthocyanin can be affected by the alterations in the seasons [21]. The anthocyanin content may relate to some factors such as temperature, pH, and the presence of phenols and metals [22].

Furthermore, the usage of anthocyanins (ANCs) is supposed to be related to a reduced risk of degenerative diseases, such as cardiovascular diseases [23], cancer [24], atherosclerosis [25], and diabetes [26]. Also, in another research, it was reported that the compounds are responsible for the inhibition of *α*-amylase and *α*-glucosidase, included flavonoids, flavonol, phenolic acid, and anthocyanins [27].

On the basis of scientific documents, this may be the first time that the antidiabetic property of anthocyanin, which is isolated from *Berberis integerrima* Bunge fruits, was investigated.

We may accredit the hypoglycemic property of the anthocyanins to their hemiacetal transformation and/or chalcone compounds.

In addition, in another research, it was reported that blueberry fruits have plenty of anthocyanin compounds, and their anthocyanins have the potential to relieve symptoms of hyperglycemia in diabetic mice [26].

Other research reported that treatment by gavage (500 mg/kg body wt) with both the blueberry extract enriched with phenolic and the fraction enriched with anthocyanin decreased raised blood glucose levels by 33 and 51%, respectively, when combined with Labrasol. This hypoglycemic property was similar to that of metformin, known as an antidiabetic drug [26].

The results of other studies proposed that mulberry fruit anthocyanin extract showed significant antidiabetic activities by controlling blood glucose levels in rats as an animal model of type 2 diabetes [28, 29]. In addition, it was reported that in vitro, *Calendula officinalis* fruit anthocyanin increased the release of insulin from pancreatic *β*-cells [7].

In this study, after using an Amberlite column for anthocyanin purification and using the pH-differential method for determination anthocyanin [30], the amount of AFBI anthocyanin increased from 14.36 ± 0.33 mg/g to 34.51 ± 0.42 mg/g (*P* value <0.0001).

It was reported that anthocyanins in purple and red flesh purple potato tubers would have significant inhibitory effects on *α*-glucosidase and *α*-amylase [30]. One approach to the management of complications of diabetes is to inhibit *α*-amylase and *α*-glucosidase activities. In the present study, we evaluated the inhibition of *α*-amylase and *α*-glucosidase by BIBF extract and anthocyanin isolated from BiB fruits.

**Figure 1:**
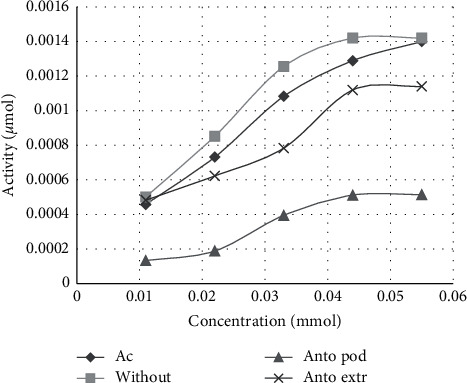
Michaelis–Menten curves of *α*-glucosidase inhibition by anthocyanin extract and purified anthocyanin in comparison to reaction without the inhibitor and acarbose as a standard. Antoextr, anthocyanin extract; Ant pod, purified anthocyanin; without, reaction without the inhibitor; Ac, acarbose.

**Figure 2:**
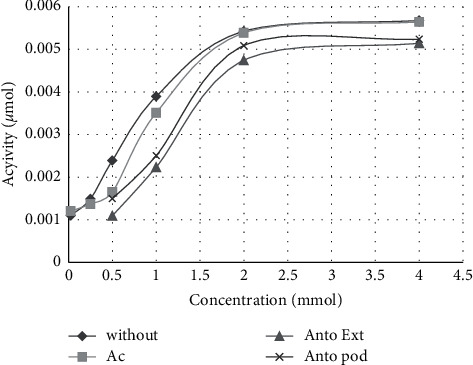
Michaelis–Menten curves of *α*-amylase inhibition by anthocyanin extract and purified anthocyanin in comparison to reaction without the inhibitor and acarbose as a standard. Antoextr, anthocyanin extract; Ant pod, purified anthocyanin; without, reaction without the inhibitor; Ac, acarbose.

Based on the obtained data, purified anthocyanin has the most efficient inhibition feature on *α*-glucosidase activity (Figures 1 and 2). In other words, the lowest IC_50_ of *α*-glucosidase inhibition was observed in purified anthocyanin of BIBF (IC_50_ = 0.71 ± 0.085 mg/ml, Table 1). The IC_50_ of BIBF extract (1 ± 0.048 mg/ml, Table 1) was less than acarbose (IC_50_ = 8.8 ± 0.14 mg/ml, *P* < 0.001, Table 1). Also, there is a significant difference between IC_50_ of purified anthocyanin and BIBF extract (*P* < 0.05), and a significant difference is observed between IC_50_ of purified anthocyanin and acarbose (*P* < 0.001).

In addition, the results of this study showed that purified anthocyanin of BIBF (IC_50_ = 1.14 ± 0.003 mg/ml, Table 1) inhibits *α*-amylase more than BIBF extract (IC_50_ = 1.4 ± 0.01 mg/ml, *P* < 0.001, Table 2), and the IC_50_ of acarbose (1 ± 0.085 mg/ml, *P* < 0.05, Table 1) was higher than purified anthocyanin of BIBF. The IC_50_ of BIBF extract (1.4 ± 0.01 mg/ml, Table 1) was less than acarbose (1 ± 0.085 mg/ml, *P* < 0.001, Table 1).

In another research, it was reported that extracts of *Rumex maderensis* exhibited a moderate *α*-amylase inhibitory potential. Flowers of *Rumex maderensis* presented the strongest inhibition, while leaves showed lowest inhibition. All mentioned samples showed higher IC_50_ values than acarbose as a commercial drug (*P* < 0.05). Also, flower extract of *Rumex maderensis* showed the most inhibition of *α*-glucosidase [31].

Based on Tables 2 and 3, the enzyme inhibition modes of purified anthocyanin (with Km = 3.03 ± 0.6 mmol and Vmax = 0.0009 ± 0.0001 *μ*mol/min for *α*-glucosidase and with Km = 0.8 ± 0.4 mmol and Vmax = 0.007 ± 0.0008 *μ*mol/min for *α*-amylase) were uncompetitive. The enzyme inhibition modes of BIBF extract (with Km = 4.54 ± 0.9 mmol and Vmax = 0.002 ± 0.0002 *μ*mol/min for *α*-glucosidase and with Km = 2.05 ± 0.57 mmol and Vmax = 0.0074 ± 0.0001 *μ*mol/min for *α*-amylase) were mixed.

It was reported that the anthocyanin extract of purple fleshed potato including petunidin, cyanidin, and malvidin compounds showed a noncompetitive inhibition in inhibition of *α*-glucosidase. In addition, Narita et al. reported that chlorogenic acid showed a mixed inhibition in inhibition of pancreatic *α*-amylase [32].

In comparison to acarbose as a competitive inhibitor, in mixed and uncompetitive inhibitions, the sample binds to broad regions of the enzyme other than the active site [33]. Another advantage of mixed inhibition is that it may not be affected by higher substrate concentrations and would still be effective at lower concentrations of the substrate [33].

## 4. Conclusion

Anthocyanin of *Berberis integerrima* fruit could inhibit *α*-glucosidase and *α*-amylase.

There are different responses to the plant extracts rich in anthocyanin, determined by certain remarkable biomarkers. The present study and similar studies provide a basis for further clinical trials. *Berberis* ssp. fruits possess different components with potential health benefits [34, 35].

## Figures and Tables

**Table 1:** IC_50_ ± SD of the enzymes inhibition of the purified anthocyanin and anthocyanin extract in comparison to acarbose.

Samples	IC_50_ of *α*-glucosidase inhibition (mg/ml)	IC_50_ of *α*-amylase inhibition (mg/ml)
Anthocyanin content after using column	0.71 ± 0.085	1.14 ± 0.003
Anthocyanin extract	1 ± 0.048	1.4 ± 0.01
Acarbose (standard)	8.82 ± 0.14	1 ± 0.085

**Table 2:** Km ± SD and Vmax ± SD and the type of *α*-glucosidase inhibition of the studied samples in comparison to acarbose.

Samples	Km ± SD (mmol)	Vmax ± SD (*µ*mol/min)	Kind of inhibition
Anthocyanin content after using column	3.03 ± 0.6↓	0.0009 ± 0.0001↓	Uncompetitive
Anthocyanin extract	4.54 ± 0.9↑	0.002 ± 0.0002↓	Mixed
Without inhibition	4.47 ± 0.01	0.0028 ± 0.0003	—
Acarbose (standard)	5.22 ± 1.04↑	0.0028 ± 0.0003 (constant)	Competitive

**Table 3:** Km ± SD and Vmax ± SD and the type of *α*-amylase inhibition of the studied samples and acarbose.

Samples	Km ± SD (mmol)	Vmax ± SD (*µ*mol/min)	Kind of inhibition
Anthocyanin content after using column	0.8 ± 0.4↓	0.007 ± 0.0008↓	Uncompetitive
Anthocyanin extract	2.05 ± 0.57↑	0.0074 ± 0.001↓	Mixed
Reaction without inhibitor	1.01 ± 0.213	0.0076 ± 0.0006	–
Acarbose (standard)	1.264 ± 0.301↑	0.0076 ± 0.0007 (constant)	Competitive

## Data Availability

The data used to support the findings of this study are available from the corresponding author upon request.
